# What Shall We Tell the People?*Read before the National Dental Association, Boston, Massachusetts, Aug. 23-27, 1920. Reprinted *Journal N. D. A.*, Aug., 1921.

**Published:** 1921-09

**Authors:** Rea Proctor M’Gee


### WHAT SHALL WE TELL THE PEOPLE?*

BY REA PROCTOR M'GEE, M.D., D.D.S.

The most difficult part of any story is the beginning. Every life is a romance, but there are few novelties because there are only a few who know where to start in the telling. Whether the audience is waiting to hear us or whether our message must draw the audience, makes little difference. If the audience waits and the message is uninteresting, there will be few listeners at the end. If there are few at the beginning and the story has the right appeal, the hall will be crowded at the finish.

If we believe that the mouth is the barometer of health, let us say so. We should tell the people that clean healthy mouths are seldom found among those who are below par physically. We should call their attention to the fact that healthy, happy rosy-cheeked children, are the children who have clean, white, serviceable teeth—not those with mouths full of decayed teeth with putrescent pulps that harbor millions of pathogenic bacteria. We should tell them that mastication is of vital importance. We have only begun to scratch the surface of the hidden truths of diet, but we know that the basic principle of nutrition is good food properly digested, and between good food and good digestion there must be thorough mastication.

---

*Read before the National Dental Association, Boston, Massachusetts, Aug. 23-27, 1920. Reprinted *Journal N. D. A.*, Aug., 1921.

The people ask, "When is the best time to begin the care of the teeth?" There is only one answer covered by one small word—*now*. *Now* is the time to begin the care of the mouth and of the teeth. Now is the time to begin any effort that is directed toward the preservation or recovery of health. Tomorrow is the enemy of health. Whatever is worth doing is worth doing *now*.

If you would put into words the splendid ideas that pass through your brain upon the hygiene of the mouth, those ideas would be doing their duty. Whenever you fail to write or speak the gospel of good teeth and good health you are doing a passive injury to your community.

All of the important things of life depend upon the health and energy of the individual. We must tell the people over and over again the danger of oral neglect. We must tell the people what the mouth does in health, what the teeth mean, how important the saliva is in the chemistry of digestion, how the saliva does its work and the meaning of mastication.

We have five special senses: sight, sound, taste, smell and touch. The sense of taste is sadly interfered with in a mouth full of cavities reeking with fermentation, a mouth pouring pus from pyorrheal pockets, a mouth with teeth and gums so sensitive that mastication is impossible, a mouth that has only enough teeth to punch holes in food, a mouth so filled with artificial substitutes that it resembles a knight in armor. Taste is the sense that instinctively causes proper mastication. It is one of the great five that were given to us for our preservation. Anything that interferes with taste interferes with digestion. Through the Eustachian tubes and the pharynx, we have a direct connection from the mouth with the sense of sound. Through the teeth the physical property of bone conduction is most acute.

The deaf are enabled to hear through bone conduction by resting a sensitized metal fan against the teeth. Is it not reasonable to believe that the sense of hearing is always less acute after the teeth are lost?

Through direct infection of the maxillary sinuses, through malposition of the teeth and through the ever-present odors of decomposition in septic mouths, the sense of smell is partially or totally lost.

The mouth bears a direct local relation to these three special senses—tasting, hearing, smelling. Among the great dangers of oral sepsis are eye complications, ranging from slight conjunctivitis to total blindness.

And the last sense of touch. The whole range of sensory nerves combine to produce the sense of touch. Is there any complication that cannot be either produced or aggravated in the sensory area by a diseased mouth?

I believe we are justified in telling the people that if they wish to preserve their five special senses, the place to start is with the mouth. The constitution of the United States guarantees to every citizen the right to the "pursuit of happiness." Is a citizen pursuing happiness with a mouth full of decayed teeth?

We should tell the people that every child has a right to demand protection from preventable disease. Almost all diseases of the mouth and teeth are preventable. They are preventable by personal and by dental care. Decay of the teeth, loss of the teeth, and the malocclusion alter or destroy facial symmetry, interfere with digestion, rob the organism of normal energy, prevent comfort, encourage disease and disrupt the orderly progress of the individual toward health, success and a long life.

The human interest in the story of dentistry can be made to grip the attention of every listener and of every reader. We have novels and plays where the heroine is beautiful but whose human interest centers in affliction. She may be blind, deaf, dumb, insane, crippled, sick, rich, poor, noble, criminal but never toothless. Society demands teeth. All of the twists and turns of the imagination will be read with interest except one. Heroes and heroines may trample upon every accepted standard except the dental standard. When they lose their teeth they must either become villians or retire.

We do not have to exaggerate to create public interest. All we have to do is to tell the story simply, plainly. The average reading intelligence is in the first year of high school. Tell the things that will hold the attention of an intelligent high-school Freshman and you have the world by the ear.

